# A Benchmark Dataset for Concealed Improvised Explosive Device Detection in X-ray Security Imaging

**DOI:** 10.1038/s41597-026-07263-7

**Published:** 2026-04-28

**Authors:** Natnael Abule Takele, Divya Velayudhan, Dwarikanath Mahapatra, Hasan AlMarzouqi, Naoufel Werghi

**Affiliations:** 1 Department of Computer Science, Khalifa University of Science and Technology, Abu Dhabi, UAE; 2 Center for Cyber-Physical Systems (C2PS), Khalifa University of Science and Technology, Abu Dhabi, UAE

## Abstract

Threat detection in X-ray security screening is critical for preventing concealed threats in airports and other high-security venue where Improvised Explosive Devices (IEDs) remain among the most persistent and dangerous threats. The lack of a representative, and publicly available IED dataset has limited the development of machine-learning based automated threat detection systems. To address these issues, we propose an open access dataset, called IEDXray constructed for automated detection of IEDs. The dataset comprises 17,360 X-ray images captured under a strategic concealment protocol, covering scenarios ranging from isolated threats to heavily cluttered baggage environments. It includes diverse IED types—homemade explosives, batteries, and modified devices such as laptops, mobile phones, pagers, and walkie-talkies. To validate the dataset, we benchmark state-of-the-art detection models, including YOLOv10, Faster R-CNN, DETR, and GroundingDINO, establishing baseline results across multiple security-screening tasks. By reflecting real-world threat concealment, clutter, and variability, IEDXray provides the research community with a high-fidelity benchmark to advance automated explosive detection and improve x-ray security screening.

## Background & Summary

X-ray based security screening is the primary modality for threat detection at airports, border checkpoints, and other high-risk public venues. While automated systems and operator training have advanced detection of conventional contraband (e.g., firearms, knives), these items are typically present as single, well-defined shapes and are therefore amenable to geometry-based recognition. In contrast, improvised explosive devices (IEDs) and electronic explosive devices that repurpose consumer electronics (laptops, phones, chargers, pagers, walkie-talkies) are highly heterogeneous, often deliberately concealed, and can closely mimic benign items, making them far more difficult to detect reliably^1–4^.

Improvised Explosive Devices (IEDs), unlike military explosives, are non-conventional explosive devices that are assembled using readily available materials^5^. IEDs are assembled from readily available components such as power source, initiator/detonator, and main charge and exhibit wide variation in size, trigger mechanism, and concealment strategy^5–7^. Historical incidents (e.g., Oklahoma City (1995), Madrid(2004), London(2005) and recent regional trends underline the continued operational significance of concealed explosives^5,7,8^. Modified consumer electronic devices are especially concerning because explosive setups are embedded within housings designed for benign consumer use, blurring the visual and material cues that standard X-ray detectors and human screeners rely upon.

Detecting IEDs differs from detecting conventional threats in several key ways. The characteristics listed below highlight why detecting IEDs presents greater complexity than common threat items.

- **Composite assemblies:** IEDs consist of multiple components—some of which are inherently benign (e.g., batteries, wires, plastic containers). This heterogeneity complicates the detection process, as individual parts may not raise alarms but collectively represent a serious threat.
- **Concealment and miniaturization:** Recent advances in miniaturization have enabled explosives to be meticulously embedded within common electronic devices such as laptops, chargers, or phones. This embedding blurs the distinction between harmless consumer electronics and potentially dangerous devices. From an X-ray imaging perspective, the visual and material profiles of such items overlap significantly, creating a high risk of false negatives^9^.
- **Dismantled components:** IEDs may also appear in dismantled forms, with their components dispersed across the bag. Individually, these components resemble innocuous objects, but together they constitute a complete explosive system. Detecting such dismantled threats requires not only small-object detection but also part-to-whole reasoning to infer suspicious co-occurrences in cluttered baggage scenes.
- **Visual ambiguity:** Current dual-energy X-ray machines can classify objects broadly into organic, inorganic, and metallic categories. However, they cannot reliably distinguish between benign organic substances (e.g., food, toiletries) and explosive materials used in IEDs. For instance, dynamite can resemble a bar of marzipan^10^. This limitation makes it particularly challenging to discriminate illicit materials hidden within everyday items, especially when adversaries deliberately exploit such weaknesses.

Current threat item detection relies heavily on manual screening baggage scans by trained operators^11^. While effective in many settings, manual screening is labor intensive, prone to human error due to operator fatigue, particularly in high-throughput airport or freight screening environments^10^. The reported recall rate for manual screening is 80–90 percent^12^, and costly to scale; training security personnel can require months of CBT (Computer Based Training) sessions to reach proficiency^13^. Automated methods based on machine learning and deep learning show promise in detecting common prohibited items^14,15^. However, automated explosive threat detection is at an early stage mainly constrained by the scarcity of large, publicly available datasets that capture the diversity and concealment strategies of IEDs. Existing popular X-ray datasets such as SIXray^16^, GDXray^17^, PIDray^18^, and OPIXray^19^, predominantly target conventional prohibited items and do not adequately represent the heterogeneity of IEDs or the subtleties of modified electronic explosives. Public datasets for IED detection are scarce^20–22^, and a few datasets that contain explosive samples include only a limited number of IED instances, making them inadequate for training reliable detectors^23,24^. In light of explosive detection, key characteristics of existing X-ray datasets are summarized in Table 1.

**Table 1:** Comparison of X-ray security inspection datasets.

Dataset	#Images	#Classes	Explosive Data	Electronic Devices	Captions	Detection Tasks	Availability
SIXray^16^	1,050,302	6	No	No	No	1	Yes
GDXray^17^	8,150	3	No	No	No	1	Yes
PIDray^18^	47,677	12	No	No	No	1	Yes
OPIXray^19^	8,885	5	No	No	No	1	Yes
CLCXray^46^	9,565	12	No	No	No	1	Yes
PIXray^47^	5,046	15	No	No	No	1	Yes
HiXray^48^	45,364	8	No	Yes	No	1	Yes
XrayPI^25^	2,409	7	Yes	No	No	1	Yes
EDS^49^	14,219	10	No	Yes	No	1	Yes
LPIXray^50^	60,950	18	No	Yes	No	1	No
STCray^24^	46,642	21	Yes	No	Yes	1	Yes
IEDXray^33^	17,360	9	Yes	Yes	Yes	3	Yes

"Explosive Data” indicates the presence of explosive-related samples, “Electronic Devices” denotes datasets containing annotated electronics (e.g., laptops, phones), and “Detection Tasks” refers to the number of supported detection problems. IEDXray supports three tasks: electronic device detection, generic explosive detection, and specific explosive detection.

Early AI-based efforts have primarily relied on small or simulated datasets, limiting their generalizability. Initial CNN-based approaches^21,25^ applied transfer learning on small-scale X-ray datasets, achieving promising but restricted classification-only performance without localization. Later work^20^ leveraged DCGAN-based augmentation to expand limited samples, reporting high accuracy but introducing synthetic bias and dataset narrowness. Texture-based feature methods with SVMs^9^ similarly faced poor scalability, long processing times. More recent studies^26^ investigating concealed circuits in laptops were constrained by narrow datasets derived from repeated imaging of a small number of devices, raising concerns about imbalance and spurious correlations. Collectively, these studies highlight that while progress has been made, explosives detection research remains heavily dataset-constrained, and insufficiently validated for operational deployment. This highlights the need for a dataset that portrays real-world screening challenges such as occlusions, overlapping objects, and variations in X-ray scan perspectives.

**Fig. 1:**
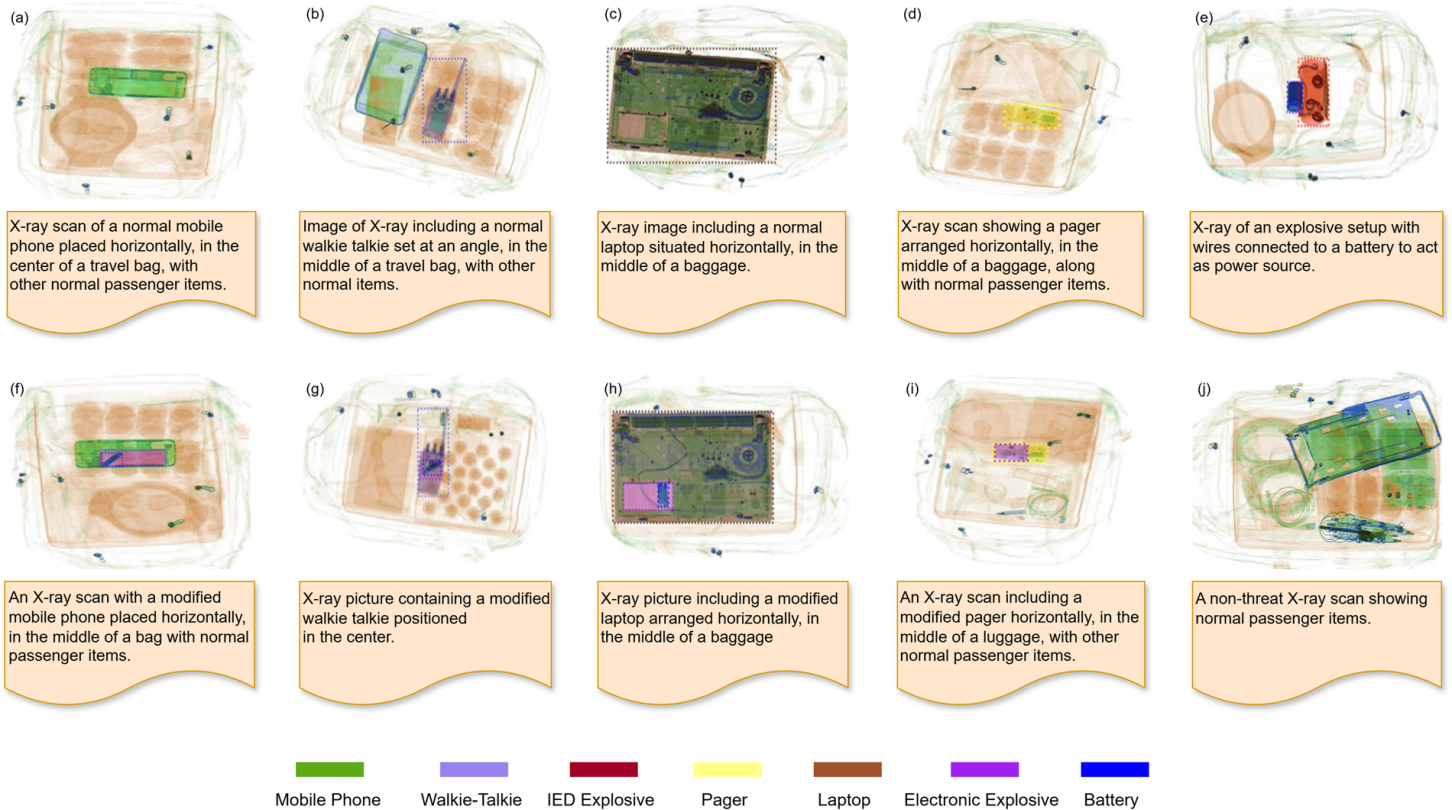
Sample images from the dataset with each object classes and textual captions. For each sample box labels are provided for the object and explosive parts in the case of electronic explosive samples.parts (**a**)-(**d**) represent normal electronic devices with no concealed electronic explosive threat. Some images have multiple objects and explosive setups illustrated in samples (**e**) to (**i**). (**j**) represents a non-threat image with no target object classes.

To address these gaps, we introduce IEDXray, a curated X-ray dataset focused on IEDs and modified electronic explosive devices. IEDXray is assembled under a strategic concealment protocol that varies clutter, occlusion, and concealment methods across realistic security screening scenarios. The dataset includes isolated and highly cluttered samples, annotated with bounding boxes for both host objects (e.g., laptop) and embedded explosive components. Representative samples are shown in Fig. 1. Our IEDXray dataset exhibits:

- **Realistic Screening Scenarios:** IEDXray encompasses both isolated and cluttered baggage samples, reflecting real-world security screening conditions. The dataset includes separately scanned electronic devices in a tray, cabin baggage, and checked luggage to capture diverse operational contexts.
- **Diversity of Threat Items:** The dataset contains IEDs and modified electronic explosives disguised as everyday objects commonly encountered in passenger belongings, such as laptops, mobile phones, beverage bottles, and batteries. This diversity in item type, shape, and size enhances the realism and variability of the threat representations.
- **Standardized Format:** All data are pre-processed and provided in the widely used COCO format, with predefined training and testing splits to ensure reproducibility and facilitate benchmarking across different detection models.

To assess the dataset’s impact, we benchmark state-of-the-art detection models on IEDXray, highlighting common failure modes and providing a reproducible baseline for future research.

## Methods

### Improvised Explosive Devices Samples

In our dataset various configurations of IEDs and their subsets, modified electronic explosives were considered. The samples used for the construction of our dataset are inert and pose no explosive threat. In order to build such inert IED and inert electronic explosive samples, we utilized harmless and readily available simulants of common explosives. Inert explosive simulants are non-hazardous compounds or mixture of compounds that effectively produce an x-ray correct signal^27^. Such simulants are identified primarily based on their closeness of their effective atomic number, Zeff and density, in g/cm3 to real explosives variants^20,27,28^. The effective atomic number is calculated using the density and mass absorption coefficients of elements for a given wavelength or photon energy^29^. For example, ammonium nitrate based low density explosives have 0.8–1 g/cm3 density and approximate effective atomic number of 7^28^. For such explosives, inert simulants like dark brown sugar(dense sugar with a high content of molasses) and flour are used in preparing inert IED training kits^28^. Based on this similarity, a simulant can be selected to simulate explosive as they can generate x-ray correct visual representation of explosives. In our dataset, dark brown sugar was used as a simulant for dynamite (primarily composed of ammonium nitrate) due to its comparable effective atomic number and X-ray attenuation properties.

Utilizing inert explosive simulants, various IED configurations were assembled^30^. The dataset includes varied samples of homemade IEDs in terms of circuit setup, packaging, shape, and size. These inert explosives are made using non-hazardous simulants visually mimicking real IEDs in x-ray security imaging. The dataset includes modified electronic explosives, a subset of IEDs utilizing laptops, mobile phones, walkie-talkies, and pagers to house explosive materials. Concurrently, the dataset includes unmodified versions of these common electronics presenting an important benchmark for anomaly detection tasks between explosive electronics and non-threat electronics. The modifications of electronics often involve removing internal components, principally the battery to create space for explosives or utilizing pre-existing gaps within the device^3,31^. The dataset includes multiple samples of such modified electronics, aiming to replicate real-world threats. Inclusion and exclusion criteria were defined to ensure the dataset’s relevance for real-world security screening applications. X-ray scans were included if they contained IEDs or components like home-made explosives (HME), modified electronics, and battery-based IEDs. Exclusion criteria eliminated fully occluded IEDs beyond detection feasibility, commercial/military explosives, poor-quality images (noise, distortions), and ambiguous annotations. All contributors were informed that the X-ray scans would be openly shared to advance automated IED detection research.

### Proposed IEDXray Dataset

To build the IEDXray dataset, images were acquired using an ANER K8065 dual-energy X-ray scanner (100–160 kV, 0.4–1.2 mA) with a tunnel size of 800 × 650 mm and sub-millimeter pixel resolution^32^.The scanner operates in its standard configuration and provides pseudo-colored RGB renderings generated internally by the system software. Raw dual-energy outputs (e.g., DICOS files or 16-bit sensor-level arrays) were not accessible during data acquisition and are therefore not included in the released dataset. The dataset includes samples of IEDs, modified electronic explosives, and non-threat items. A strategic scanning protocol was developed to define various levels of concealment for different IED configurations. This protocol for simulating common concealment strategies used in real-world scenarios, such as those found in baggage screening. In addition, the IEDXray dataset captures the visual, shape, size and compositional diversity of IEDs while reflecting real-world concealment, clutter, and screening scenarios. A detailed description of the data collection process is presented in the following sections.

#### Strategic Threat Concealment

**Fig. 2:**
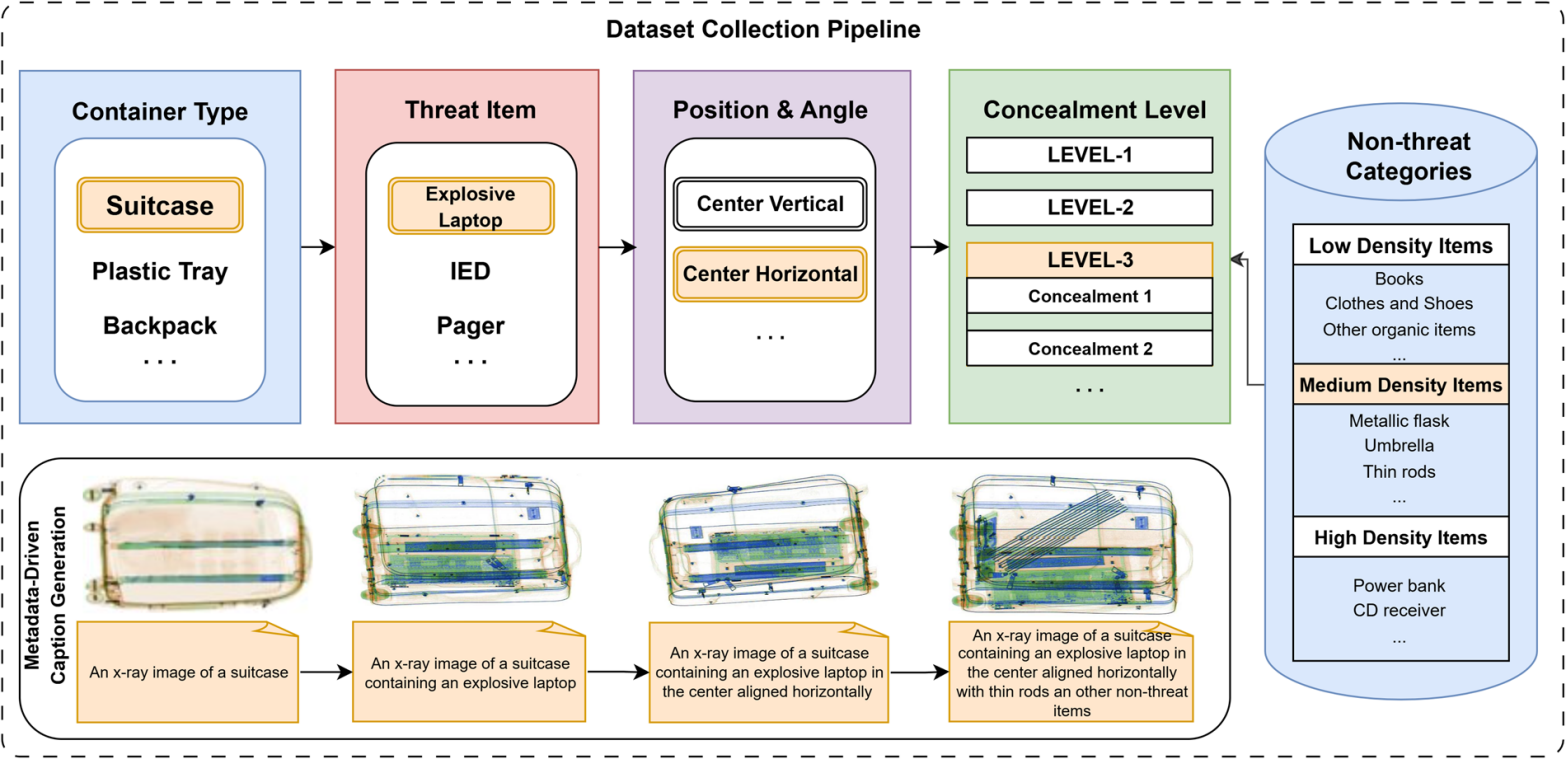
Metadata-driven protocol for IEDXray dataset construction, selecting baggage type, threat item, position, and orientation to systematically simulate concealment scenarios and support caption generation.

The IEDXray dataset is constructed using a meta-data that defines strategic concealment designed to replicate realistic adversarial tactics. We designed the dataset collection pipeline, shown in Fig. 2, ensuring diversity in both concealment and scanning conditions to support the development of models with strong generalization. Two key elements define this protocol. First, the protocol incorporates three scanning scenarios that reflect common security practices: open tray scans, cabin baggage scans, and checked baggage scans. Second, a concealment spectrum was introduced to simulate increasing complexity in threat concealment levels, ranging from fully visible cases to highly cluttered and occluded scenarios. Along this spectrum, IED samples were placed in two distinct positions and three orientations to generate varied perspectives of the same threat item. To illustrate the range, representative stages are described as follows:

- **Fully Visible:** The threat item is unobstructed and clearly identifiable.
- **Light Concealment:** The item is partially covered by a small number of benign objects, causing minimal obstruction.
- **Moderate Concealment:** The item is intermixed with several benign items, introducing noticeable clutter and partial occlusion.
- **Heavy Concealment:** The item is surrounded by dense benign objects, creating significant overlap and reduced visibility.
- **Highly Concealed:** The item is located within clutter, with strong occlusion and only limited features visible.

Lastly, the protocol defines that the IED is deliberately positioned opposite the X-ray source, ensuring occlusion by benign items and maximizing concealment complexity. This design yields a dataset that captures broad variation in clutter, viewing angles, and occlusion, providing a realistic benchmark for automated explosive detection systems.

#### Data Annotation

All images generated under the concealment protocol were annotated through a structured pipeline. Annotation quality was ensured through multiple verification steps: (1) targeted annotator training: A team of annotators underwent targeted training, and (2) guideline-driven labeling: annotators followed detailed labeling guidelines to ensure accuracy and consistency during the annotation process. Annotators were provided with metadata derived from the protocol to guide the identification of bounding boxes for both threat items and host objects. (3) independent review for consistency: A separate review team verified all annotations to eliminate inconsistencies or errors. In addition, redundant annotations were filtered. This annotation pipeline ensures high-quality ground truth suitable for training and evaluating automated detection methods.

#### Dataset Split Strategy

The dataset was partitioned at the physical IED configuration level. Images derived from the same assembled threat device and concealment setup were assigned exclusively to either the training or test split. Although similar container types (e.g., suitcases or trays) may appear in both splits, each scan corresponds to a separately acquired physical configuration. For each image, benign objects were manually arranged under distinct positioning, concealment levels, and object layouts. As a result, scene composition, object placement, and threat assemblies differ across splits.

**Fig. 3:**
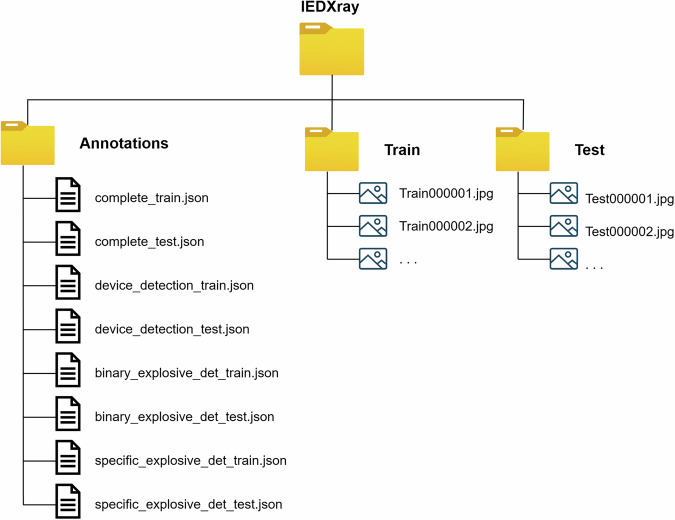
Folder Structure of the IEDXray Dataset.

**Fig. 4:**
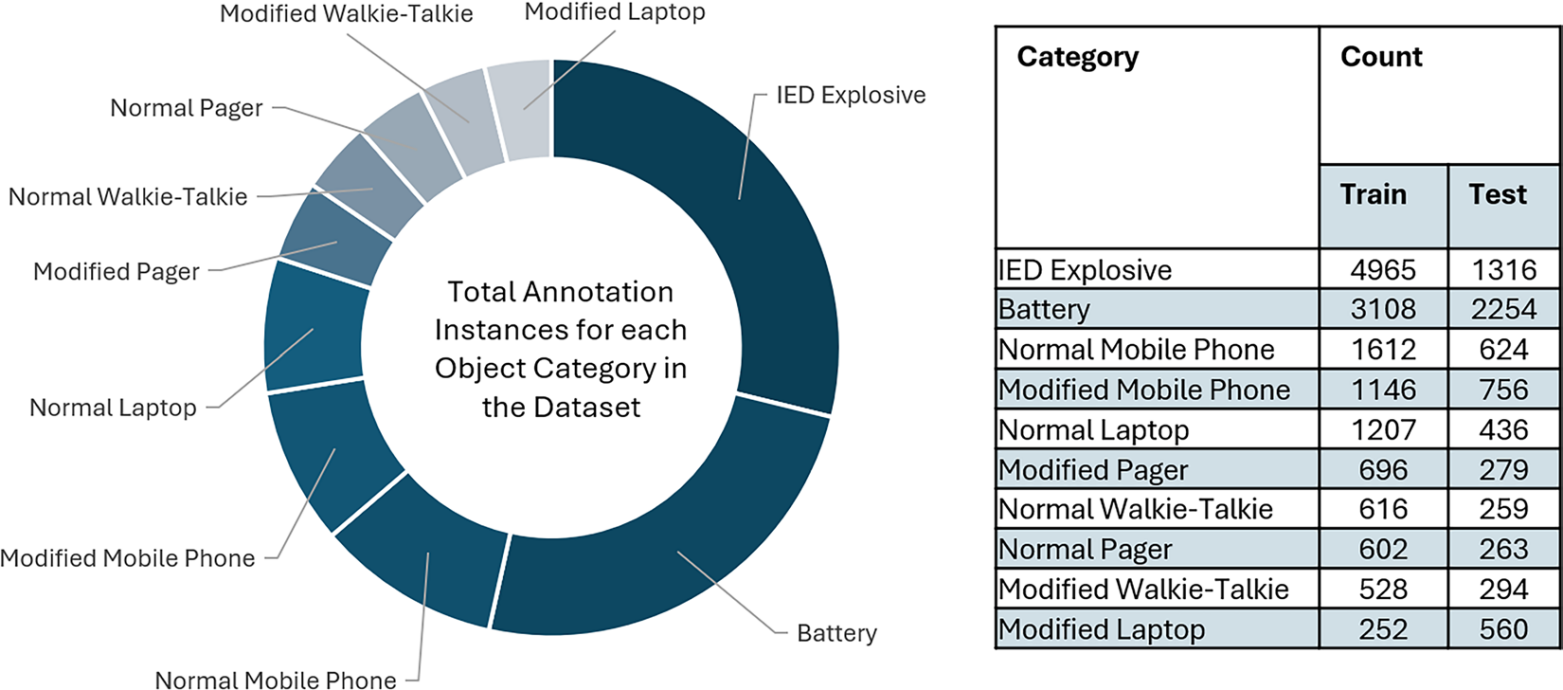
Total annotation instances of object classes in the explosive dataset.

This sample-wise partitioning leads to non-uniform class distributions between training and testing. In particular, certain modified electronic explosive configurations appear more frequently in the test split refer to Fig. 4. This design was intentional to evaluate model generalization on unseen assemblies and more challenging concealment scenarios.

## Data Records

The IEDXray^33^ dataset is publicly available at 10.6084/m9.figshare.30784328. with a total of 17,360 xray images. To facilitate usability, the dataset has been formatted in accordance with the COCO standard and structured into two primary splits, *train* directory *test* directory, and annotations directory, located under the root directory, IEDXray, as illustrated in Fig. 3. The *annotations* directory provides eight distinct COCO-standard JSON files corresponding to the specific detection task and dataset split. These cover four annotation file pairs for train and test — complete detection that contains all object annotations, ground-truth labels for bounding boxes, and textual caption describing each x-ray image. While the device detection annotation file contain ground truth boxes of electronic devices only. The third annotation group is dedicated for specific explosive detection containing box annotation for specific categories explosives. The fourth set of annotation contains annotations group of boxes for all explosives as one group. This design ensures that while all tasks share the same image data, the annotations are modular and can be independently utilized for training, evaluation, or task-specific model benchmarking.

## Data Overview

The dataset contains a total of 17,360 X-ray image split into 12,224 train and 5,136 test images. It encompasses multiple object categories relevant to IED detection, including batteries, explosive containers, explosive components, laptops, mobile devices, and complete IED assemblies. As shown in Fig. 4, the distribution of annotation instances across categories is imbalanced, reflecting the frequency of appearance of different components. In IEDXray dataset, many images contain multiple annotated objects simultaneously, such as an explosive laptop scan has annotations for batteries and its modified region and the laptop itself. This provides a detailed set of information for detecting suspicious electronic explosive configurations in complex, cluttered baggage environments where multiple suspicious items often co-occur.

## Technical Validation

We evaluate state-of-the-art (SOTA) detection models on the proposed IEDXray dataset to benchmark their performance. The evaluation is designed around tasks that simulate scenarios relevant to X-ray security checkpoints. These experiments provide a comparative reference for developing and assessing IED detection algorithms using IEDXray dataset.

### Baseline Detection Models

We benchmark a diverse set of state-of-the-art (SOTA) object detection models to validate the usability and challenge level of the IEDXray dataset. The evaluation includes CNN-based detectors such as YOLOv10^34^, Faster R-CNN^35^, Cascade R-CNN, RetinaNet^36^, FCOS^37^, RTMDet^38^, and TOOD^39^, representing one-stage, two-stage, anchor-based, anchor-free, and task-aligned detection paradigms.

We further include transformer-based detectors such as DETR^40^ and Grounding DINO^41^, as well as X-ray domain-specific models including SDANet^42^,and AO-DETR^43^. This broad selection ensures coverage across multiple architectural families and design philosophies, providing a comprehensive benchmark for explosive detection in X-ray security imaging.

#### Implementation Details

The detection models were implemented using the MMDetection framework, except where official repositories were used for YOLOv10 (from Ultralytics), AO-DETR, and SDANet. Models were initialized with pre-trained weights finetuned on IEDXray. All models employed a ResNet-50 (R50) backbone unless otherwise specified. Grounding DINO used a Swin Transformer backbone, and the YOLOv10-M variant was used for YOLO. Training was conducted using the default optimization parameters provided in the official MMDetection configuration with the default learning rate schedule defined in each model’s configuration file. Standard data augmentation strategies provided by the framework were applied, including random horizontal flipping and multi-scale resizing. No additional task-specific augmentation techniques were introduced.

All experiments were trained on the IEDXray training split and evaluated on the corresponding test split using the standard COCO evaluation API. Model complexity metrics, number of parameters (in millions), GFLOPs, and inference speed (FPS), are reported in the benchmarking tables. We use RTX4090 GPU 24GB VRAM for training and report inference speed(FPS) with batch size 1 under FP32 inference.

#### Evaluation Metrics and Protocol

To benchmark our dataset on detection models, we rely on evaluation metrics widely used in benchmark datasets such as COCO. Specifically, we report **Mean Average Precision (mAP@[0.5:0.95]),** **Average Precision at IoU = 0.5 (AP50)**, and **Average Recall (AR@[0.5:0.95])** to evaluate performance under different scenarios.

##### Mean Average Precision (mAP@[0.5:0.95])

Calculated as the mean of Average Precision (AP) over multiple Intersection-over-Union (IoU) thresholds ranging from 0.50 to 0.95 in steps of 0.05. This metric captures model performance across both lenient and strict matching criteria and serves as the primary benchmark for object detection.

##### Average Precision at IoU = 0.5 (AP50)

Measures precision at a single IoU threshold of 0.5, corresponding to the PASCAL VOC metric^44^, and is often used for comparison with prior X-ray detection studies.

##### Average Recall (AR@[0.5:0.95])

Computed as the mean recall across IoU thresholds from 0.50 to 0.95 in steps of 0.05, this metric reflects a detector’s ability to identify all ground-truth instances, which is particularly critical in X-ray security imaging where missed detections carry high operational risk.

**Fig. 5:**
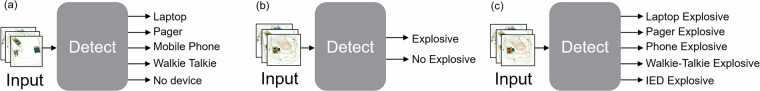
Illustration of the three detection tasks in our dataset: (**a**) electronic device detection, (**b**) generic explosive detection, and (**c**) specific explosive detection.

Using the above metrics, we benchmark our dataset on detection models based on scenarios from x-ray baggage security screening. These scenarios include detecting IED threats, detecting electronic devices in baggage scans, and screening electronic devices for IED modifications or explosive setups. From these scenarios, we have setup the tasks below for benchmarking SOTA models on detection. For each of the three tasks shown in Fig. 5, we finetuned state-of-the-art detection models using the corresponding subset of the IEDXray dataset. The dataset annotation file was filtered for each task to match the task definition:

- **Electronic Device Detection:** This task assesses the ability of detection models to identify and classify electronic devices within baggage scans, as shown in Fig. 5a. Labels were restricted to electronic device categories (e.g., laptop, mobile phone, pager, walkie-talkie), reflecting common security procedures where certain devices must be screened separately at checkpoints.
- **Generic Explosive Detection:**In this task, models are required to detect any explosive object or setup within a baggage scan. Detecting IED threats is particularly challenging for security personnel due to the wide variation in their shape, size, type, and configuration. For benchmarking, all explosive threats and their variants were consolidated into a single class, and models were trained to detect their presence regardless of explosive type, or explosive setup shown in Fig. 5b.
- **Specific Explosive Detection:** In this task, models are evaluated on their ability to distinguish between different types of explosive devices. Specifically, the goal is to determine whether an IED setup belongs to one of five categories—laptop, pager, mobile phone, walkie-talkie, or homemade IED—as illustrated in Fig. 5c. This presents a unique challenge, as models must also differentiate normal, non-threat electronics from their modified or explosive counterparts. For training, labels were assigned to the five explosive categories, while benign electronics were treated as negative examples.

### Quantitative Benchmark Results

In this section, we report quantitative evaluation results of the benchmarked detection models after fine-tuning on the IEDXray training split. Table 2 presents the results for the electronic device detection task, alongside model complexity metrics for reference. Across models, detection performance varies depending on architecture and design choices. Grounding DINO achieves an AP of 69.0 and AR of 71.9, while YOLOv10-M achieves an AP of 67.0 with higher inference speed (77 FPS). Other models, including Faster R-CNN, Cascade R-CNN, DETR, and RTMDet, exhibit a range of AP and AR values, reflecting differences in detection and localization behavior under cluttered baggage conditions.

**Table 2:** Comparison of detection models on electronic device detection in IEDXray.

Model	Input	Params (M)	GFLOPs	FPS	AP	AP50	AR
YOLOv10-M^34^	640 × 640	16.5	63.99	77.0	67.0	**73.4**	70.8
Faster R-CNN^35^	800 × 896	41.4	187	21.7	54.7	69.3	66.6
Cascade R-CNN^51^	800 × 896	69.2	214	22.0	58.5	69.6	66.6
SDANet^42^	800 × 896	64.8	180	20.2	55.3	68.2	59.5
Grounding DINO^41^	640 × 640	172	464	8.4	**69.0**	72.0	71.9
DETR^40^	800 × 896	72.1	234	10.2	49.6	60.1	60.5
AO-DETR^43^	640 × 640	58.4	26.9	14.1	59.0	64.4	63.6
RetinaNet^36^	800 × 896	36.4	184	26.9	58.2	70.1	66.9
FCOS^37^	800 × 896	32.1	181	26.1	56.4	70.0	64.4
RTMDet^38^	640 × 640	24.7	39.1	28.5	61.3	68.2	**92.2**
TOOD^39^	800 × 896	32.0	177	13.9	64.0	72.6	76.0

Model complexity metrics (Input, Params, GFLOPs, FPS) are reported for reference. In the table, AP represents mAP@[0.5:0.95], and AR refers to AR@[0.5:0.95].

Two-stage detectors such as Faster R-CNN and Cascade R-CNN show moderate performance, while transformer-based models generally exhibit higher recall, particularly in densely packed scenes. Notably, RTMDet achieves high AR values, indicating strong object coverage by proposing objects from the X-ray scan scene in the dataset. However, moderate AP scores are observed due to differences in localization precision and false positive behavior.

Building upon the electronic device detection results, we next evaluate models on the generic explosive detection task. This task is more challenging, as all explosive variants are consolidated into a single class and often appear under heavy occlusion and clutter. As shown in Table 3, Grounding DINO scores an AP of 48.9 and an AP50 of 67.6 in identifying diverse explosive configurations. RTMDet attains an AR of 61.1, indicating object coverage in the IEDXray dataset^33^. Transformer-based DETR and two-stage detectors such as Faster R-CNN and Cascade R-CNN exhibit moderate performance. This highlights the increased difficulty of detecting consolidated explosive categories compared to device-level detection.

**Table 3:** Comparison of detection models on generic explosive detection.

Model	mAP@[0.5:0.95]	mAP50	AR@[0.5:0.95]
YOLOv10^34^	35.1	58.0	48.4
Faster R-CNN^35^	14.9	32.4	27.5
Cascade R-CNN^51^	16.7	34.1	36.1
SDANet^42^	20.6	38.2	30.5
Grounding DINO^41^	**48.9**	**67.6**	57.2
DETR^40^	24.1	46.4	36.6
AO-DETR^43^	16.8	30.0	27.6
RetinaNet^36^	24.4	53.3	38.0
FCOS^37^	13.1	33.1	33.0
RTMDet^38^	28.9	53.7	**61.1**
TOOD^39^	15.3	36.4	40.3

Refer model complexity metrics (Input, Params, GFLOPs, FPS) in Table 1.

For the specific explosive detection task, models are required to distinguish modified explosive devices from visually similar benign electronics. As shown in Table 4, Similar to the generic detection task, performance varies across models, with AP values ranging from 11.7 to 43.5. Transformer-based models generally achieve higher recall, reflecting improved object coverage in cluttered scenes. However, precision limitations and localization challenges constrain AP improvements. Two-stage detectors such as Faster R-CNN and Cascade R-CNN exhibit comparatively lower performance, highlighting the difficulty of distinguishing modified devices from normal electronics under dense packing and miniaturization. Overall, performance across all models is lower than in the device detection task, underscoring the increased complexity of fine-grained explosive classification within realistic baggage screening conditions.

**Table 4:** Comparison of detection models on specific explosive detection.

Model	mAP@[0.5:0.95]	mAP50	AR@[0.5:0.95]
YOLOv10^34^	36.8	59.1	49.0
Faster R-CNN^35^	18.0	36.5	38.7
Cascade R-CNN^51^	16.7	32.7	35.1
SDANet^42^	13.2	22.1	20.0
Grounding DINO^41^	**43.5**	**61.2**	47.5
DETR^40^	22.5	43.6	41.9
AO-DETR^43^	20.8	35.2	43.5
RetinaNet^36^	13.6	28.7	37.4
FCOS^37^	11.7	23.7	32.4
RTMDet^38^	29.0	54.6	**61.0**
TOOD^39^	19.3	35.9	36.5

Refer model complexity metrics (Input, Params, GFLOPs, FPS) in Table 2.

### Qualitative Evaluation

Building upon the quantitative results, we provide a qualitative assessment of the aforementioned detection tasks using representative models. This evaluation helps to identify challenging cases and failure scenarios, as well as to better understand the performance differences across models on our IEDXray dataset. We present qualitative results for YOLOv10, Faster R-CNN, Cascade R-CNN, Grounding DINO, and DETR on explosive detection.

**Fig. 6:**
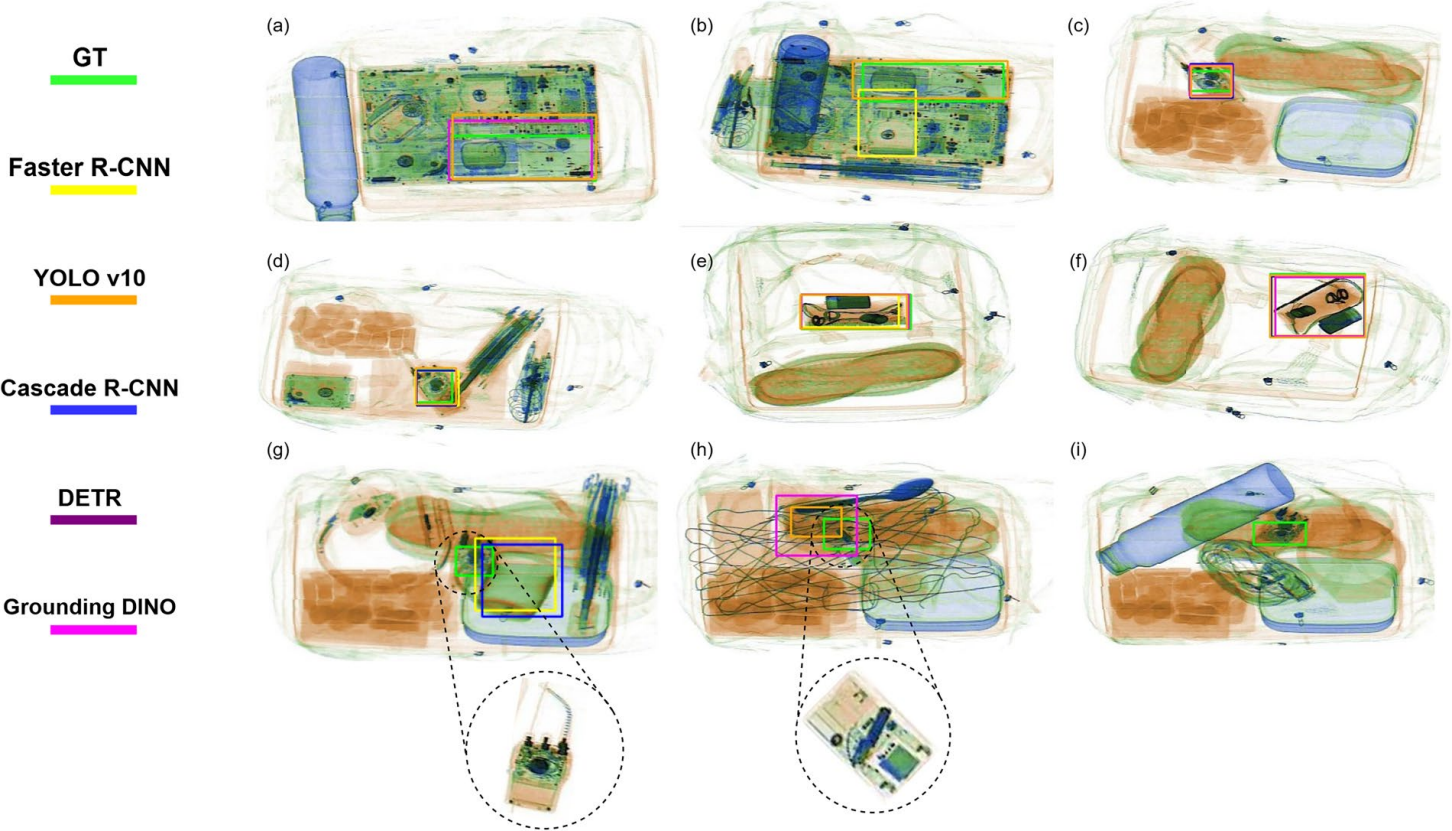
Qualitative results of binary explosive detection along with Ground Truth (GT) images. Missing boxes indicate missed detections.

Figure 6 illustrates ground truth (GT) annotations alongside the detection outputs of the different models. Grounding DINO, which achieved the best overall performance in the quantitative evaluation, also demonstrates highly accurate localization for explosives concealed within electronic devices such as laptops, and walkie-talkies, as shown in Fig. 6(a,c,d,f). Similarly, YOLOv10 achieves precise bounding box predictions for both sparse and cluttered baggage scan images. Both Grounding DINO and YOLOv10 further demonstrate strong capability in detecting miniature explosive setups embedded within small electronic devices, such as pagers and walkie-talkies in Fig. 6(g,h).

In contrast, other models such as Cascade R-CNN, DETR, and Faster R-CNN produce less accurate bounding box overlaps with respect to the ground truth. Figure 6(i), for example, shows a missed detection in a scan containing a walkie-talkie with a concealed explosive. This highlights the challenges posed by varying levels of baggage clutter and the miniaturization of threat items present in IEDXray dataset.

The qualitative evaluation reveals that while Grounding DINO and YOLOv10 achieve strong localization performance, baggage X-ray imagery presents persistent challenges. Concealment of explosives within electronic devices reduces discriminative cues, making miniature setups in compact devices such as pagers and walkie-talkies particularly difficult to detect. In addition, background clutter and overlapping items in scans frequently interfere with bounding box predictions, leading to drift or misalignment in models such as Cascade R-CNN, DETR, and Faster R-CNN. Missed detections are observed when threat items are heavily occluded or miniaturized. Detection failures can largely be attributed to limited appearance information for small or uniformly textured threats, the presence of clutter and occlusion, and the compounding effect of multiple challenges within a single scan. These findings underscore the complexity of automated explosive threat detection and emphasize the need for robust models capable of handling concealment, scale variation, and interference. They also highlight the importance of IEDXray to serve as a realistic dataset in advancing research and development in security screening domain.

### Limitations

Despite its scale and diversity, The IEDXray dataset also exhibits limitations due to controlled data acquisition conditions, which is limited by the diversity of scanner hardware, energy spectra, image resolutions, and operational environments represented. The dataset is collected from a single ANERk0865 dual-energy X-ray scanner (100–160 kV, 0.4–1.2 mA) which provides processed RGB images. The images are generated by a dual-energy spectra and lack high resolution multi-spectral images. These factors restrict the dataset’s usage in some alternative physics-based processing techniques. In addition, although IEDXray includes a range of explosive configurations, there is an uneven representation across threat types. Certain categories, particularly miniature devices such as pagers and walkie-talkies, are underrepresented or exhibit limited variation. This imbalance may affect model performance and generalization across rare or less frequent threat scenarios. These limitations should be taken into account when interpreting results and when applying models trained on IEDXray to broader operational contexts.

## Data Availability

The IEDXray dataset^33^ is available at Figshare (10.6084/m9.figshare.30784328).

## Code availability

During data curation, we used LabelMe^45^ for manual image annotation and a custom captioning pipeline to automatically generate textual descriptions for each annotated instance. To facilitate reproducibility, we also provide the fine-tuned model weights, and code scripts for the experiments conducted in this study. Most experiments were performed using MMDetection, while selected experiments used models from their official repositories. Caption generation codes and instructions for loading the models, running inference, and reproducing results are included in the github repositoryhttps://github.com/Natnael-gh/IEDXray.
